# Contemporary Chemotherapy Benchmarks for Molecularly Defined Advanced NSCLC: Reconstructed Individual Patient-Level Data of 22 Randomized Trials

**DOI:** 10.1007/s00408-026-00946-3

**Published:** 2026-10-07

**Authors:** Deborah Di-Xin Zhou, Sarah J. Lord, Frank Lin, Thomas John, Robert John Simes, Chee Khoon Lee

**Affiliations:** 1 Sydney Clinical Trials Centre, University of Sydney, Level 6, Lifehouse, 119-143 Missenden Rd, Camperdown, NSW 2050 Australia; 2 The Daffodil Centre, University of Sydney, Camperdown, NSW Australia; 3 Garvan Institute of Medical Research, Darlinghurst, NSW Australia; 4 Peter MacCallum Cancer Centre, 305 Grattan St, Melbourne, VIC Australia

**Keywords:** NSCLC, Actionable genomic alterations, Chemotherapy outcomes, Reconstructed individual patient data

## Abstract

**Purpose:**

The increasing adoption of targeted therapies over chemotherapy has made randomized controlled trials (RCT) with chemotherapy increasingly challenging in advanced NSCLC with actionable genomic alterations (AGA), particularly for rare subgroups. Contemporary chemotherapy outcomes specific to AGAs are lacking despite growing reliance on single-arm studies and external-control analyses. We sought to establish first-line platinum-doublet chemotherapy outcomes in advanced NSCLC with AGAs using data from RCTs.

**Methods:**

We extracted objective response rate (ORR), progression-free survival (PFS), and overall survival (OS) data from Phase 2/3 RCTs. Individual patient-level data were reconstructed from Kaplan–Meier curves to pool and compare PFS and OS across AGAs.

**Results:**

Twenty-two RCTs (*n *= 2132) were included. Across most AGAs, platinum-doublet chemotherapy produced similar outcomes-pooled ORRs ranging from 28.7 to 39.0% and median PFS from 6.0 to 7.3 months. Pairwise comparisons showed better ORR/PFS for *RET* fusion (one trial with control arm-chemotherapy/pembrolizumab) and shorter OS for *KRAS* mutation versus other AGAs (logrank *P*<.001). Detailed pooled ORR and median PFS and OS were: *ALK* rearrangement (*n* = 461)—ORR 39.0% (95% CI 25.8–52.2), PFS 7.0 months (6.8–7.2), OS 43.0 months (32.2-inestimable); common *EGFR* mutation (*n* = 1038)—ORR 28.7% (22.3–35.1), PFS 6.0 months (5.7–6.3), OS 27.4 months (26.1–29.3); uncommon *EGFR* mutation (*n* = 61)—ORR 35.1% (12.6–57.7), PFS 6.7 months (5.2–8.4), OS 29.5 months (13.3—inestimable); *EGFR* exon 20 insertion (*n* = 330)—ORR 38.3% (21.7–55.0), PFS 7.3 months (6.9–8.6), OS 30.1 months (29.1—inestimable); *KRAS* mutation (*n* = 140)—ORR 21.7% (12.4–30.9), PFS 6.2 months (4.7–6.9), OS 10.8 months (8.8–12.7); *RET* fusion (*n* = 102)—ORR 65.0% (54.5–75.5), PFS 11.4 months (8.9–16.9), OS not available.

**Conclusion:**

Platinum-doublet chemotherapy yields remarkably consistent ORR and PFS across most advanced NSCLC with AGAs. These data provide contemporary molecularly defined benchmarks that may support interpretation of single-arm studies, development of external-control analyses, and future trial design in this rare population.

**Supplementary Information:**

The online version contains supplementary material available at https://doi.org/10.1007/s00408-026-00946-3.

## Introduction

Non–small cell lung cancer (NSCLC) is a biologically heterogeneous malignancy characterized by distinct oncogenic driver mutations that define its clinical behavior and response to targeted therapies. Historically, platinum-based chemotherapy was the standard first-line treatment for advanced NSCLC, until the discovery of actionable genomic alterations (AGAs) [1] that fundamentally transformed its management. While targeted therapies have substantially improved outcomes for patients with common alterations such as *EGFR* mutations and *ALK* rearrangements, the natural history of many other molecular subgroups—particularly those lacking effective targeted options—remains poorly defined. For example, patients harboring rarer AGAs, such as *HER2* mutations or *BRAF* V600E mutations, have been traditionally included in biomarker-unselected cohorts, with no separate evaluation of their prognostic profiles and responses to therapy.

As increasingly potent targeted therapies are developed for rare genomic subgroups of NSCLC, conducting head-to-head randomized controlled trials (RCT) against standard chemotherapy has become both logistically challenging and ethically constrained. Consequently, external or historical control data are critical to interpret efficacy signals observed in single-arm or early-phase studies [2]. However, most current benchmarks for targeted therapy trials are derived from biomarker-unselected chemotherapy populations. For example, phase 2 trials such as CODEBREAK 100 [3] and DESTINY Lung-02 [4], investigating *KRAS* G12C tyrosine kinase inhibitor (TKI) and *HER2* antibody–drug conjugate respectively, benchmarked their efficacy against the chemotherapy arm of the biomarker-unselected REVEL trial [5]. Yet emerging evidence indicates that chemotherapy efficacy can vary substantially across molecular subgroups. In first-line settings, patients with *RET* fusions have demonstrated objective response rates (ORRs) of 65% and median progression-free survival (PFS) of 11.2 months, compared with 25% and 5.7 months, respectively, among those with *KRAS* G12C mutations [6, 7].

Establishing these benchmarks provides a critical foundation for interpreting the performance of emerging targeted therapies when evidence is derived from single-arm studies; particularly when randomized comparisons are impractical due to the rarity of these molecular subsets. To address this need, we characterized outcomes among patients with newly diagnosed advanced NSCLC harboring specific AGAs who received standard platinum-based chemotherapy in RCTs.

## Methods

### Eligibility Criteria

We identified published phase 2 or 3 RCTs that included treatment naive adult patients with advanced NSCLC harboring a pre-specified AGA who received first line platinum-based doublet chemotherapy and reported data for one or more outcomes (ORR, PFS, overall survival [OS]). Eligible AGAs were *EGFR* mutation (common, uncommon and exon 20 insertions), *KRAS* G12C mutation, *MET* exon 14 skipping mutation, *ALK* fusion, *ROS1* fusion, *HER2* mutation, *BRAF* V600E mutation, *RET* fusion, *NTRK* fusion and *NRG1* fusion. These alterations were selected because they are recognized in the National Comprehensive Cancer Network (NCCN) Guidelines as established or emerging molecular drivers with available targeted therapeutic options [8]. *EGFR* mutations were further divided into *EGFR* common (defined by classical sensitizing mutations such as exon 19 deletion and exon 21 L858R that predict strong response to *EGFR* TKIs), uncommon (defined by rarer *EGFR* mutations with variable TKI sensitivity) and exon 20 insertion (defined by in frame insertions or duplications within exon 20 which are generally resistant to *EGFR* TKIs) mutations. Studies were included if they reported data for at least 25 participants in the chemotherapy control arm with an eligible AGA subgroup, and published Kaplan–Meier survival curves with corresponding risk tables to allow reconstruction of individual participant data (IPD).

### Search Strategy

We performed a systematic search of the Cochrane Central Register of Controlled Trials (CENTRAL) and MEDLINE, and reviewed reference lists of included studies published between January 2000 and June 2025. The search strategy was structured around two key components: trial-related terms and cancer population descriptors (see Supplementary Table 1 for search strategies).

### Data Collection and Analysis

For each study, we extracted data from the chemotherapy arm only. These data included: study names and characteristics (phase of study and year of first recruitment), AGAs, baseline characteristics (targeted agent, number of patients, age, gender, smoking status, region [Asian vs Global], stage, ECOG status, histology), and types of platinum-based regimens. We recorded the number of objective responses. We extracted survival data coordinates from published Kaplan–Meier curves using the WebPlotDigitizer [9]. IPD were reconstructed via a validated web-based *Shiny* application built on the *IPDfromKM* R package, which accounts for censoring [10]. Quality checks were performed by comparing reconstructed data with original data. This included a summary of median PFS (95% confidence interval [CI]) as reported by the original studies and their corresponding reconstructed values. Risk of bias was assessed using the Cochrane Risk of Bias tool with domain 2 (bias due to deviations from the intended interventions) and domain 4 (bias in measurement of the outcome) most relevant for our planned cross study subgroup comparisons [11].

ORRs to chemotherapy were calculated as the proportion of patients with complete or partial responses among those with RECIST-evaluable disease. Within each AGA subgroup, ORRs from individual studies were pooled using a random-effects, inverse-variance–weighted model. Tests of interaction were performed to evaluate differences in ORR across AGA subgroups. Heterogeneity between trials and between subgroups was assessed using the *I*^*2*^ statistic (low: 0–40%, moderate: 30–60%, substantial: 50–90%; considerable: 75–100%). [12] Pre-specified subgroup analysis was performed to examine pooled ORR for different AGAs according to trials conducted in Asia vs global regions and according to pemetrexed vs non-pemetrexed based chemotherapy. For each AGA subgroup, reconstructed PFS data for patients treated with chemotherapy from individual trials were pooled. PFS distributions were summarized with Kaplan–Meier estimates and compared across AGA subgroups using the log-rank test. Hazard ratios (HRs) and 95% CI were estimated using Cox proportional hazards models. Tests of interaction were performed to evaluate differences in PFS across AGA subgroups. The same analytic approach was applied to OS.

All statistical tests were two-sided, and a nominal significance level of 0.05 was used.

## Results

**Fig. 1:**
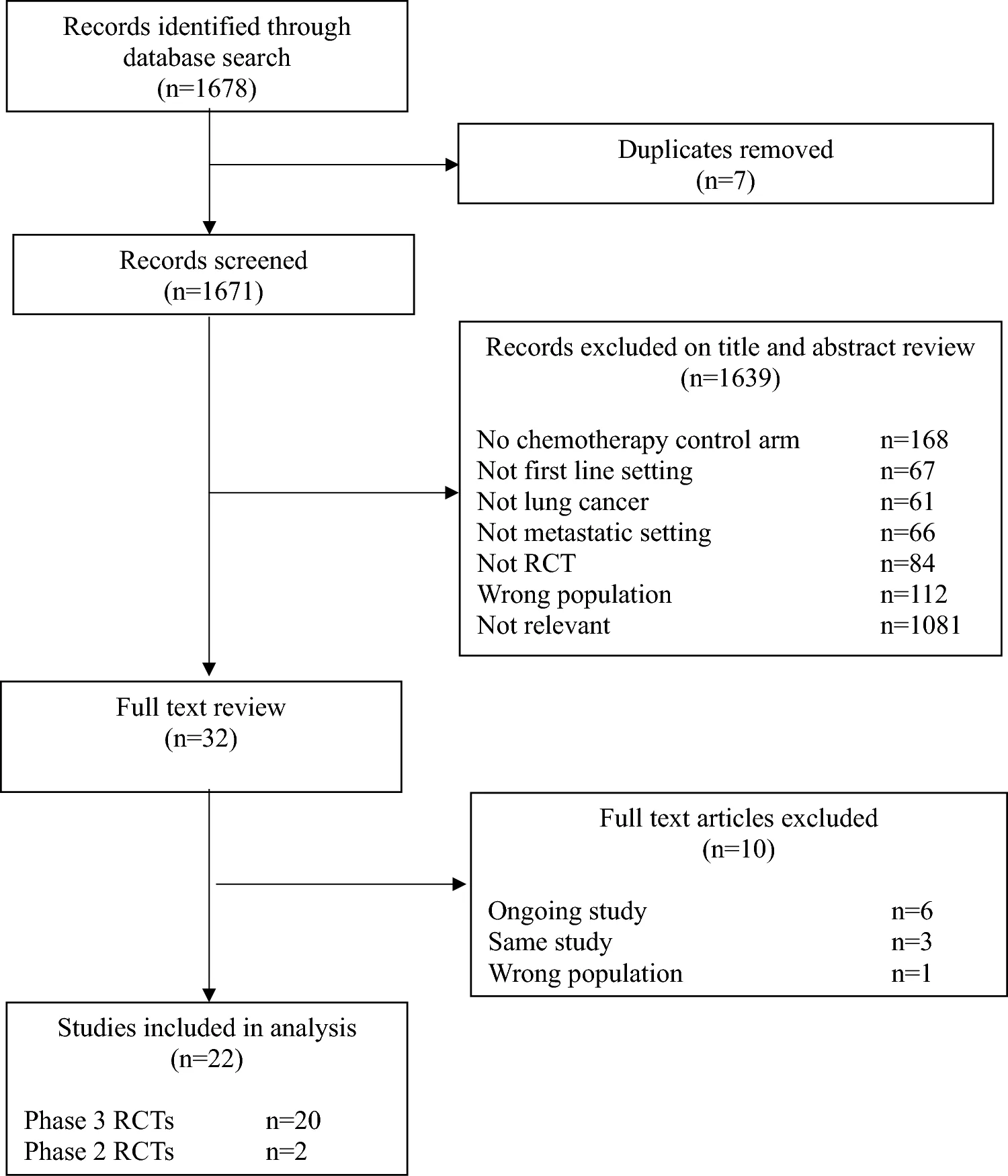
**PRISMA Flow diagram**RCT, randomized controlled trials

We found 22 eligible advanced NSCLC RCTs which reported first-line chemotherapy outcomes in 2132 participants with AGAs (Fig. 1) [6, 13–35]. Study characteristics are summarized in Supplementary Table 2. The large majority were phase 3 studies, with two phase 2 studies [20, 30]. The number of participants in the eligible chemotherapy control arms ranged from 25 to 187. First-line chemotherapy regimens consisted of a platinum agent (cisplatin or carboplatin) combined with a second agent (gemcitabine, pemetrexed, docetaxel, or paclitaxel). In LIBRETTO 431, 81% of the control arm were treated with chemotherapy and pembrolizumab [6]. In IMPOWER 150, the control arm included chemotherapy and bevacizumab [32]. Fifteen trials (68%) were classified as some concern for risk of bias due to deviations from intended interventions (due to trial context) and outcome measurement (due to lack of blinding) (Supplementary Table 3). Median PFS (95% CI) as reported by the original studies and their corresponding reconstructed values are summarized in Supplementary Table 4.

### Objective Response Rate

**Fig. 2:**
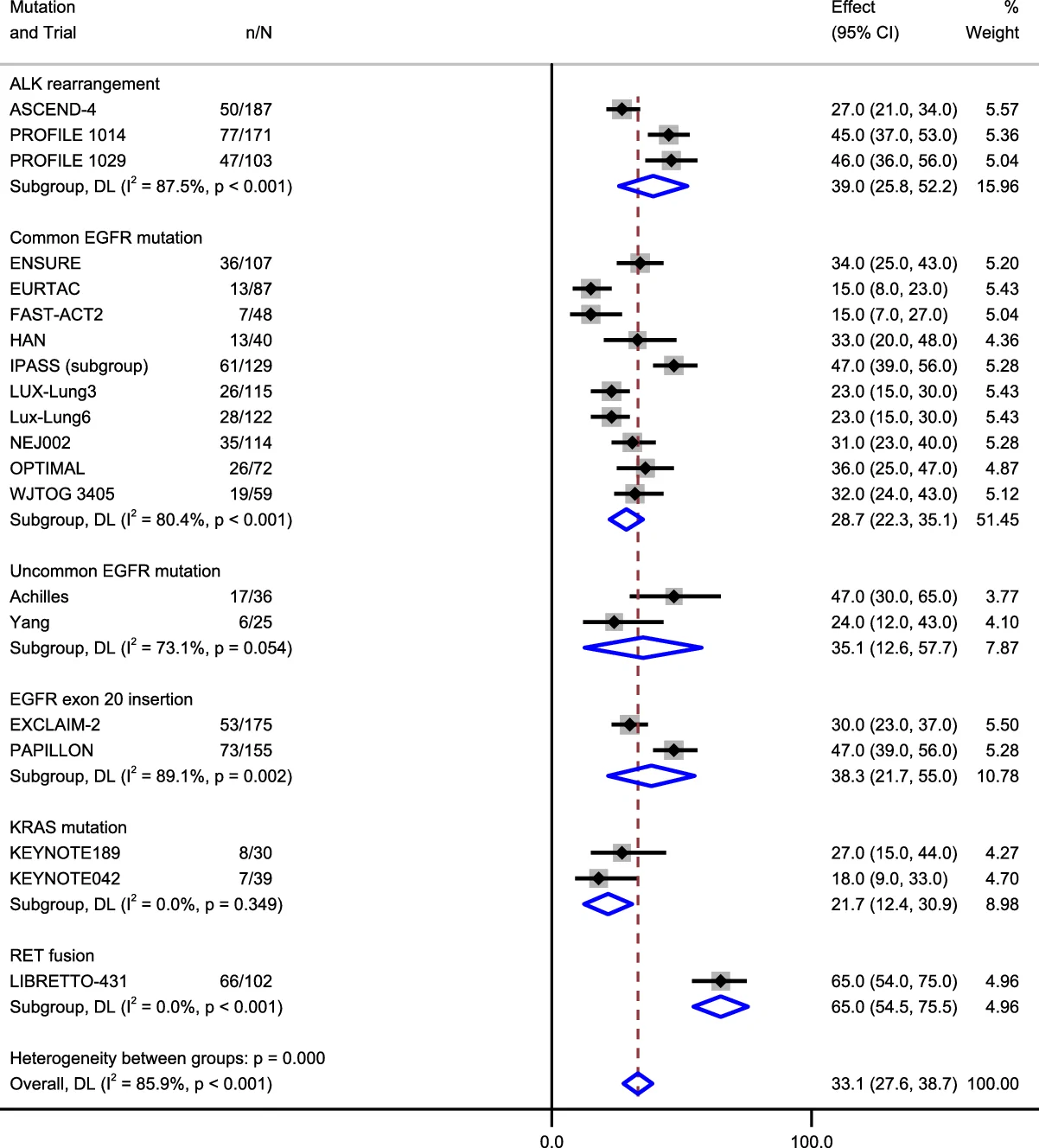
**Objective response rates pooled by actionable genomic alteration, 20 studies. ***ALK*, anaplastic lymphoma kinase; *EGFR*, epidermal growth factor receptor; *KRAS,* kirsten rat sarcoma viral oncogene homolog, *RET*, rearranged during transfection

Twenty trials reported ORR (1916 participants with measurable disease). The number of trials reporting data for each AGA subgroup ranged from 10 trials for common *EGFR* mutation (893 participants) to one trial for *RET* fusion (102 participants). The pooled ORR for AGA subgroups ranged from 21.7% (95% CI 12.4–30.9) for *KRAS* mutation to 65% (95% CI 54.5–75.5) for *RET* fusion (Fig. 2). The overall pooled ORR was 33.1% (95% CI 27.6–38.7). Overall, heterogeneity between AGA subgroups was substantial (I^2 ^= 85.9%, *P* < 0.001). For subgroups with data available from more than one trial, there was also moderate-substantial heterogeneity in ORR between trials within AGA subgroups with the exception of *KRAS* mutation.

Numerical differences in pooled ORR between AGA subgroups were not statistically significant in pairwise comparisons. The exception was *RET* fusion which was statistically significantly higher than all other subgroups (excluding *EGFR* exon 20 insertion) (*P *< 0.05; Supplementary Table 5a).

No differences in pooled ORR were observed by region (Asian vs global) for *ALK* rearrangement, common *EGFR* mutation or uncommon *EGFR* mutation (Supplementary Fig. 1). Pooled ORR did not differ for common *EGFR* mutation between pemetrexed and non-pemetrexed chemotherapy trials (Supplementary Fig. 2).

### Progression-Free Survival

**Fig. 3:**
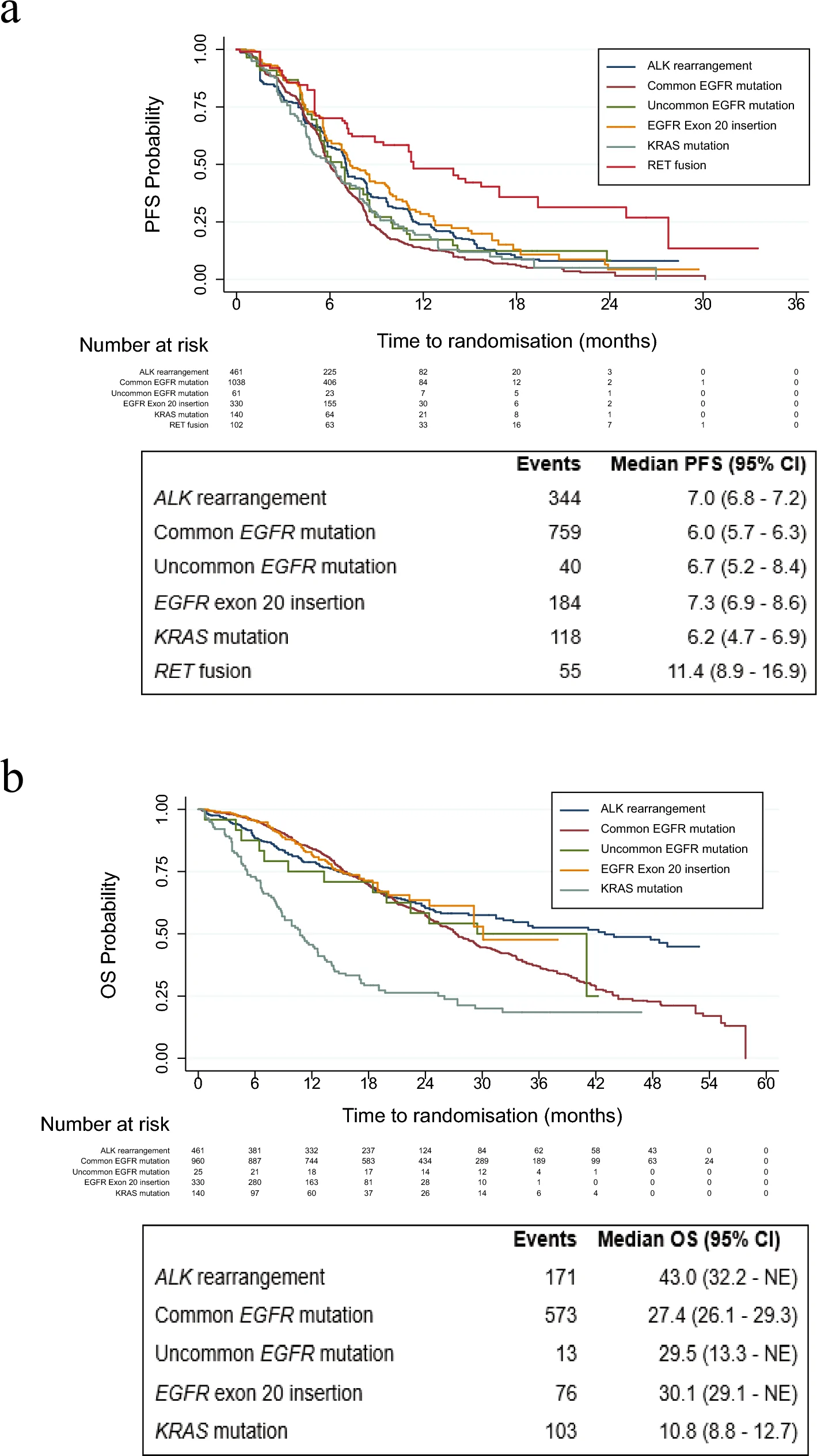
**Reconstructed a) progression-free survival (22 studies) and b) overall survival (19 studies) pooled by actionable genomic alteration. ***ALK*, anaplastic lymphoma kinase; *EGFR*, epidermal growth factor receptor; *KRAS,* kirsten rat sarcoma viral oncogene homolog, NE not evaluable; *RET*, rearranged during transfection

Twenty-two trials reported PFS (2132 participants). The pooled median follow-up was 13.9 months with a total of 1500 (70%) events. The pooled PFS for AGA subgroups ranged from 6.0 months (95% CI 5.7–6.3) for common *EGFR* mutation to 11.4 months (95% CI 8.9–16.9) for *RET* fusion (Fig. 3a). The pooled median PFS was 6.7 months (95% CI 6.4–6.9).

Numerical differences in pooled PFS between AGA subgroups were not statistically significant in pairwise comparisons. The exceptions were: *ALK* rearrangement versus common *EGFR* mutation (median PFS: 7.0 versus 6.0 months, logrank *P* < 0.001), common *EGFR* mutation versus *EGFR* exon 20 insertion (6.0 versus 7.3 months, logrank *P* < 0.001), *KRAS* mutation versus *EGFR* exon 20 insertion (6.2 versus 7.3 months, logrank *P* = 0.004) and *RET* fusion versus all other AGA subgroups (*P* < 0.001) (Supplementary Table 5b).

### Overall Survival

Nineteen trials reported OS (1915 participants). The pooled median follow-up was 29.6 months with a total of 936 (49%) events. The pooled OS for AGA subgroups ranged from 10.8 months (95% CI 8.8–12.7) for *KRAS* mutation to 43.0 months (95% CI 32.2-not evaluable) for *ALK* rearrangement (Fig. 3b). The pooled median OS was 27.4 months (95% CI 26.1–29.2).

Numerical differences in pooled OS between AGA subgroups were not statistically significant in pairwise comparisons. The exceptions were *KRAS* mutation which had statistically significantly shorter OS than all other subgroups (*P* < 0.006 and *P* < 0.001), and *ALK* rearrangement versus common *EGFR* mutation (median OS 43.0 versus 27.4, *P* < 0.003) (Supplementary Table 5c).

### Post-Hoc Sensitivity Analyses

**Fig. 4:**
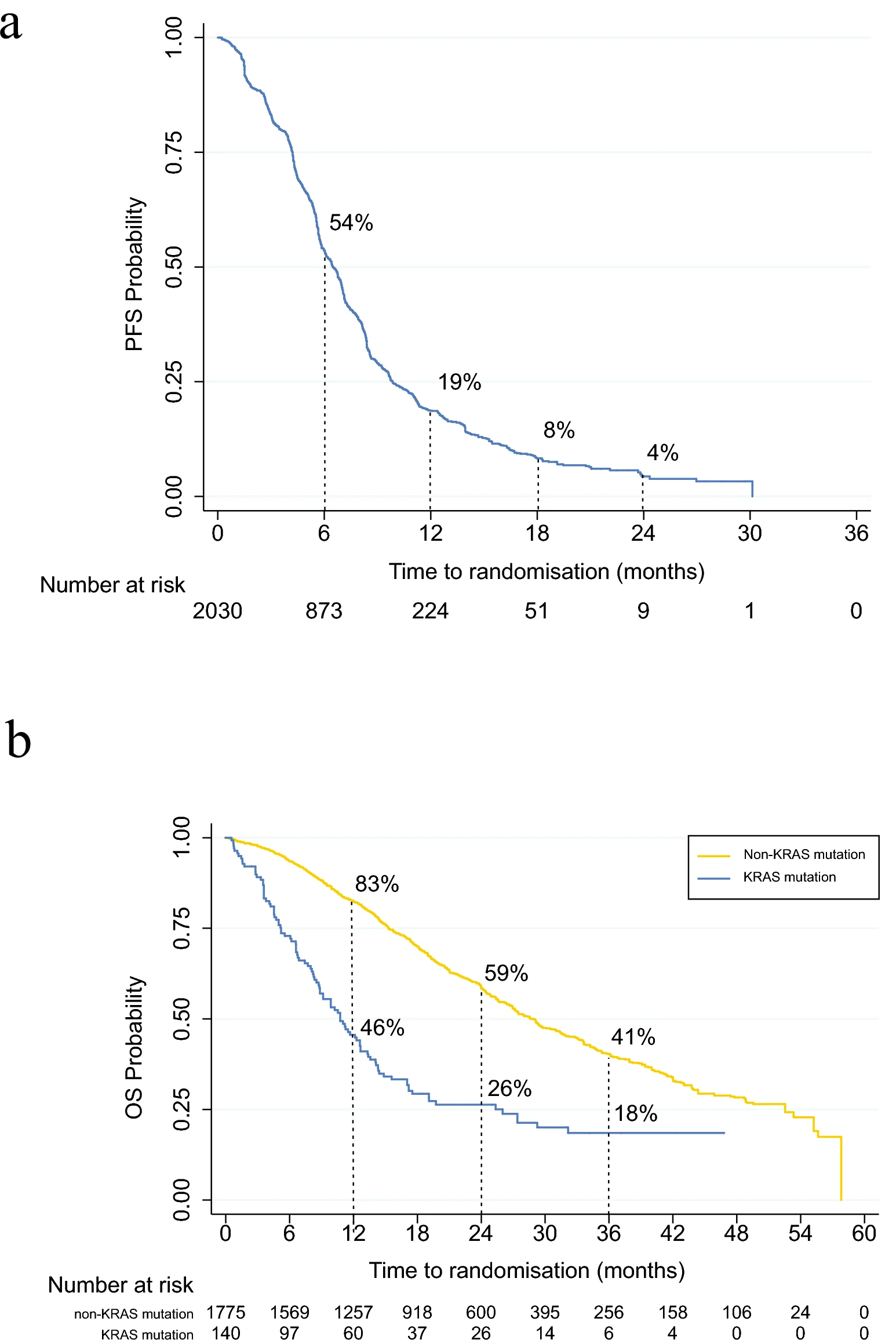
**a) Progression-free survival (PFS) by actionable genomic alteration excluding *RET* fusion (21 studies) and b) Overall survival (OS) comparing *KRAS* mutations versus other actionable genomic alterations (*ALK* rearrangement, common *EGFR* mutation, uncommon *EGFR* mutation, and *EGFR* exon 20 insertion). ***ALK*, anaplastic lymphoma kinase; *EGFR*, epidermal growth factor receptor; *KRAS,* kirsten rat sarcoma viral oncogene homolog, *RET*, rearranged during transfection

As LIBRETTO 431 appeared to be an outlier [6], we performed a post-hoc analysis excluding *RET* fusions which demonstrated a pooled ORR of 31.8% (95% CI 26.8–36.8). Median PFS was 6.5 months (95% CI 6.1–6.8) with 6-month PFS of 54%, 12-month PFS of 19%, 18-month PFS of 8% and 24-month PFS of 4% (Fig. 4a).

Since *KRAS* mutation was associated with significant shorter OS, we performed a post-hoc analysis separating *KRAS* mutation from other AGAs (*ALK* rearrangement, common and uncommon *EGFR* mutation, and *EGFR* exon 20 insertion). The median OS for *KRAS* mutation compared to non-*KRAS* mutation was 10.8 months (95% CI 8.8–12.7) and 29.1 months (95% CI 27.2–31.1) respectively. 12-month OS for *KRAS* mutation was 46%, 24-month PFS was 26% and 36-month PFS was 18% (Fig. 4b).

## Discussion

We evaluated outcomes in patients with newly diagnosed advanced NSCLC harboring AGAs who received platinum-based doublet chemotherapy. Responses and survival outcomes were similar with modest numerical differences across AGA subgroups. *KRAS* mutation appeared to be an exception with poorer survival whilst there is uncertainty regarding the improved outcomes in *RET* fusion. To our knowledge, this represents the most comprehensive synthesis of chemotherapy outcomes of AGAs to date, supported by data from over 2100 participants across twenty-two RCTs.

Overall, we found that outcomes for *ALK* rearrangements, common *EGFR* mutations, uncommon *EGFR* mutations, and *EGFR* exon 20 insertions subgroups were similar, while *KRAS* mutation and *RET* fusion subgroups appeared different. The *KRAS* mutation subgroup demonstrated shorter OS outcomes compared to other AGA subgroups, despite having similar PFS outcomes. This discrepancy may reflect differences in baseline poor prognostic characteristics associated with *KRAS* mutation or variations in post-progression therapies—details which were unavailable [7, 33]. These findings are consistent with previous clinical trials and retrospective studies reporting median PFS of 4.2–6.6 months and median OS of 10.6–16 months [36–39]. Together, this suggests that chemotherapy survival outcomes for *KRAS* mutations are inferior to those of other subgroups. For *RET* fusions, outcomes were estimated from one trial, where the control arm included pembrolizumab with chemotherapy [6]. This likely contributed to the higher ORR and longer PFS observed. Retrospective studies suggest that patients with *RET* fusion treated with only doublet chemotherapy typically experience ORRs of 45–51% and median PFS of 7.8–19 months [36, 40–42]. Taken together, this raises uncertainty regarding whether these outcomes for *RET* fusion are superior to those of other subgroups.

Our study provides estimates of average outcomes that reflect the baseline trajectory of disease under standard chemotherapy. Characterizing the true natural history of AGAs in advanced NSCLC is challenging as withholding effective treatment is ethically unacceptable. Previously, knowledge about prognosis under chemotherapy is inferred from biomarker agnostic trials. For example, in the pivotal KEYNOTE−189 [43] trial investigating immunotherapy plus chemotherapy versus chemotherapy alone in patients with newly diagnosed metastatic NSCLC without *ALK*-, *EGFR*- or *ROS1*- alterations, the median PFS was 4.9 months and median OS was 10.7 months. In contrast, we demonstrate that patients with newly diagnosed advanced NSCLC harboring an AGA who received standard platinum-based doublet chemotherapy achieved a median PFS of 6.7 months and median OS of 27.4 months. This suggests that patients with AGA have a more favorable baseline disease biology even before targeted therapy. These findings are important to facilitate patient-clinician discussions for example regarding expected prognosis compared to those without AGA or between AGA subgroups and contextual comparison with targeted therapies.

These findings have important implications for interpretation of early-phase targeted therapy trials and clinical trial design. Deriving benchmark outcomes for new trials from previous biomarker agnostic studies may be inappropriate and potentially misleading when evaluating therapies in biomarker-defined populations, as underlying disease biology and treatment responsiveness may differ [2, 44]. Using the previous KEYNOTE−189 [43] trial results—we can see there is a risk of inflated treatment effects when controls are benchmarked from biomarker agnostic trial results. Use of inaccurate assumptions can adversely affect sample size calculation and ability to detect true treatment effects in clinical trials, potentially leading to false negative or false positive findings, and ultimately trial failure [45, 46].

By leveraging prospective clinical trial data rather than real-world or retrospective datasets, our study provides a more reliable benchmark for trial populations. Investigators may use these AGA-specific estimates to inform sample size calculations and define appropriate treatment estimands; potentially enabling trials in rarer genomic subgroups to be performed without control arms. This approach may improve the likelihood of trial success—as efficacy outcomes from early phase single-arm or non-randomized trials have been shown to translate reliably to larger RCTs in lung cancers defined by AGAs [47, 48].

This study also has important research implications as several clinical gaps remain. No randomized controlled data exists for *NTRK*, *MET* exon 14 skipping, *BRAF* V600E, *HER2* mutations and *NRG1* mutations. Beyond informing clinical trial design, the AGA-specific benchmarks generated in this study can serve as external comparators for real-world datasets or registry-based observational studies. Future prospective studies and experimental validation will be valuable to confirm the generalizability and clinical applicability of these benchmark outcomes. Finally, the methodological approach used here—reconstructing individual patient-level data from published Kaplan–Meier curves—can be applied to evaluate outcomes of AGA treated with other modalities, such as antibody–drug conjugates, when individual patient-level data is unavailable.

This study has several strengths. We pooled high quality data from twenty-two RCTs providing greater power to assess chemotherapy outcomes across AGA—an approach necessary given the rarity of some alterations. There are also limitations. The lack of individual patient-level data for baseline, tumor and molecular characteristics prevented further analysis and multivariable adjustment for key prognostic characteristics. Reconstruction of data from digital extraction may not fully capture censoring or granular survival data—however the reconstructed data was comparable to trial results. Some *KRAS* [7, 32, 33] and *EGFR* [19, 21, 31] trials included were post-hoc analyses and may have introduced selection bias due to imbalanced populations. Lack of blinding in certain trials may lead to bias in outcome assessment where investigations may cease control treatment earlier leading to shorter PFS. Timing of radiological assessments also differed between trials. Survival assessment may also be impacted by post-progression therapies.

In conclusion, this study provides the most comprehensive synthesis of chemotherapy outcomes across AGA subgroups in newly diagnosed advanced NSCLC, leveraging high-quality data from RCTs. These findings can provide critical benchmarks for interpretation of early phase targeted trials, design of future clinical trials and contextualization of patient-clinician discussions.

## Supplementary Information

Below is the link to the electronic supplementary material.

Supplementary file1 (DOCX 69 KB)

## Data Availability

Data extracted from included studies and used for analyses are publicly available.
