# Supplementary material for: Contemporary Chemotherapy Benchmarks for Molecularly Defined Advanced NSCLC: Reconstructed Individual Patient-Level Data of 22 Randomized Trials
Source: Lung. 2026 Oct 7;204(1):83. doi: 10.1007/s00408-026-00946-3 (PMC13645942; doi:10.1007/s00408-026-00946-3)
Supplement: Supplementary file 1 [file 408_2026_946_MOESM1_ESM.docx]

**Contemporary chemotherapy benchmarks for molecularly defined advanced NSCLC: reconstructed individual patient-level data of 22 randomized trials**

**Supplementary Figures and Tables**

**Journal: Lung**

**Author list:**

Deborah Di-Xin Zhou

Sarah J. Lord

Frank Lin

Thomas John

Robert John Simes

Chee Khoon Lee

**Corresponding author:** Dr Deborah Di-Xin Zhou - deborah.zhou@sydney.edu.au

**Supplementary Table 1: Search strategy, CENTRAL and MEDLINE**

| 1 | Anaplastic Lymphoma Kinase |
| --- | --- |
| 2 | ALK.mp |
| 3 | EML4-ALK.mp |
| 4 | Receptor, ErbB-2 |
| 5 | HER2.mp |
| 6 | Exon 20.mp |
| 7 | KRAS.mp |
| 8 | “Proto-Oncogene Proteins p21(ras)” |
| 9 | Egfr.mp |
| 10 | Epidermal growth like factor.mp |
| 11 | Anaplastic lymphoma receptor tyrosine kinase.mp |
| 12 | Receptor Protein-Tyrosine Kinases |
| 13 | Molecular Targeted Therapy |
| 14 | Oncogenes |
| 15 | Carcinoma, Non-Small-Cell Lung |
| 16 | Nsclc.mp |
| 17 | Lung cancer*.mp |
| 18 | Lung carcinoma*.mp |
| 19 | Lung Neoplasms |
| 20 | Lung tumor*.mp |
| 21 | Lung tumour*.mp |
| 22 | Non small cell*.mp |
| 23 | Nonsmall cell*.mp |
| 24 | 15 or 16 or 17 or 18 or 19 or 20 or 21 or 22 or 23 |
| 25 | Randomized Controlled Trial |
| 26 | Controlled Clinical Trial |
| 27 | Randomized.mp |
| 28 | Randomly.mp |
| 29 | Clinical trial |
| 30 | 25 or 26 or 27 or 28 or 29 |
| 31 | (Drug Therapy or Drug Therap* or Chemotherap* or Pharmacotherap*).mp. [mp=title, book title, abstract, original title, name of substance word, subject heading word, floating sub-heading word, keyword heading word, organism supplementary concept word, protocol supplementary concept word, rare disease supplementary concept word, unique identifier, synonyms, population supplementary concept word, anatomy supplementary concept word] |
| 32 | (Antineoplastic Agents or Antineoplastic* or Antitumo* or Anticancer).mp. [mp=title, book title, abstract, original title, name of substance word, subject heading word, floating sub-heading word, keyword heading word, organism supplementary concept word, protocol supplementary concept word, rare disease supplementary concept word, unique identifier, synonyms, population supplementary concept word, anatomy supplementary concept word] |
| 33 | 31 or 32 |
| 34 | Proto-Oncogene Proteins c-ret/ |
| 35 | Proto-Oncogene Proteins B-raf/ |
| 36 | Proto-Oncogene Proteins c-met/ |
| 37 | 1 or 2 or 3 or 4 or 5 or 6 or 7 or 8 or 9 or 10 or 11 or 12 or 13 or 14 or 34 or 35 or 36 |
| 38 | 24 and 30 and 33 and 37 |
| 39 | limit 38 to (english language and humans) |

| **Study/Author** | **Actionable genomic alteration** | **Phase** | **Year of 1^st^**  **Recruitment** | **Targeted Agent** | **Control arm** | **No. pt control arm** | **Age**  **(median, yrs)** | **Women (%)** | **Never smoker (%)** | **Region** | **Stage IV (%)** | **ECOG 0 (%)** | **Adeno-carcinoma (%)** |
| --- | --- | --- | --- | --- | --- | --- | --- | --- | --- | --- | --- | --- | --- |
| ASCEND-4 | ALK fusion | 3 | 2013 | Ceritinib | Platinum/  Pemetrexed | 187 | 54 | 114 (61) | 122 (65) | Global | 182 (97) | 70 (37) | 180 (96) |
| PROFILE 1014 | ALK fusion | 3 | 2011 | Crizotinib | Platinum/  Pemetrexed | 171 | 54 | 108 (63) | 112 (71) | Global | 168 (98) | 163 (95) | 161 (94) |
| PROFILE 1029 | ALK fusion | 3 | 2012 | Crizotinib | Platinum/  Pemetrexed | 103 | 50 | 60 (58) | 72 (70) | Asia | 96 (93) | 99 (96) | 101 (98) |
| CONVINCE | EGFR Common | 3 | 2013 | Icotinib | Platinum/  Pemetrexed | 122 | 56 | 95 (69) | 108 (79) | Asia | 122 (89) | 130 (95)^ | * |
| ENSURE | EGFR Common | 3 | 2011 | Erlotinib | Platinum/  Gemcitabine | 107 | 56 | 65 (61) | 74 (69) | Asia | 100 (94) | 15 (14) | 100 (94) |
| EURTAC | EGFR Common | 3 | 2007 | Erlotinib | Platinum/  Gemcitabine or Docetaxel | 87 | 65 | 68 (78) | 63 (72) | Global | 82 (94) | 30 (34) | 78 (90) |
| FAST-ACT2 | EGFR Common | 3 | 2009 | Erlotinib  (intercalated) | Platinum/  Gemcitabine | 48 | * | * | * | Asia | * | * | * |
| HAN | EGFR Common | 2 | 2011 | Gefitinib | Platinum/  Pemetrexed | 40 | 9  (27.5%) # | 23 (58) | 29 (72) | Asia | 33 (83) | 10 (25) | 40 (100) |
| IPASS (EGFR subgroup) | EGFR Common | 3 | 2006 | Gefitinib | Platinum/  Paclitaxel | 129 | 90  (70%) # | 103 (80) | 122 (95) | Global | * | 122 (95)^ | * |
| LUX-LUNG3 | EGFR Common | 3 | 2009 | Afatinib | Platinum/  Pemetrexed | 115 | 61 | 77 (67) | 81 (70) | Global | 98 (85) | 41 (35) | 115 (100) |
| LUX-LUNG6 | EGFR Common | 3 | 2010 | Afatinib | Platinum/  Gemcitabine | 122 | 58 | 83 (68) | 99 (81) | Asia | 116 (95) | 41 (34) | 122 (100) |
| NEJ002 | EGFR Common | 3 | 2006 | Gefitinib | Platinum/  Paclitaxel | 110 | 63& | 73 (64) | 66 (57) | Asia | 84 (74) | 57 (50) | 110 (97) |
| OPTIMAL | EGFR Common | 3 | 2008 | Erlotinib | Platinum/  Gemcitabine | 72 | 59 | 43 (60) | 50 (69) | Asia | 67 (93) | 69 (96)^ | 62 (86) |
| WJTOG 3405 | EGFR Common | 3 | 2006 | Gefitinib | Platinum/  Gemcitabine | 86 | 64 | 60 (70) | 57 (66) | Asia | 41 (48) | 52 (60) | 84 (98) |
| ACHILLES | EGFR Uncommon | 2 | 2019 | Afatinib | Platinum/  Pemetrexed | 36 | 66.5 | 20 (56) | 13 (36) | Asia | * | 16 (44) | * |
| Yang (post-hoc) | EGFR Uncommon | 3 | 2007 | Afatinib | Platinum/  Gemcitabine or Pemetrexed | 25 | 61 | 14 (56) | 21 (84) | Global | 24 (96) | 11 (44) | 25 (100) |
| EXCLAIM-2 | EGFR exon 20 insertion | 3 | 2020 | Mobocertinib | Platinum/  Pemetrexed | 175 | 62 | 116 (66) | 106 (61) | Global | 166 (95) | 74 (42) | 172 (98) |
| PAPILLON | EGFR exon 20 insertion | 3 | 2022 | Amivantamab + Platinum/  Pemetrexed | Platinum/  Pemetrexed | 155 | 62 | 93 (60) | 91 (59) | Global | * | 55 (35) | 153 (99) |
| IMPOWER 150 (post-hoc) | KRAS | 3 | 2015 | Atezolizumab + Bevacizumab/  Platinum/  Paclitaxel | Platinum/  Paclitaxel/ Bevacizumab | 71 | 62 | 27 (38) | 3 (4) | Global | * | 26 (37) | * |
| KEYNOTE 042  (post-hoc) | KRAS | 3 | 2014 | Pembrolizumab | Platinum/  Pemetrexed or Paclitaxel | 39 | * | * | * | Global | * | * | * |
| KEYNOTE 189  (post-hoc) | KRAS | 3 | 2016 | Pembrolizumab + Platinum/  Pemetrexed | Platinum/  Pemetrexed | 30 | * | * | * | Global | * | * |  |
| LIBRETTO 431 | RET fusion | 3 | 2022 | Selpercatinib | Platinum/  Pemetrexed +/- Pembrolizumab | 102 | 63 | 45 (44) | 68 (67) | Global | 93 (82) | 40 (39) |  |

**Supplementary Table 2: Baseline characteristics of studies and patients**

*Not reported

^Combined ECOG 0 and 1 reported

\# Age calculated ≥65

& Mean

**Supplementary Table 3: Risk of Bias**

| **Trial** | **Domain 1** | **Domain 2** | **Domain 3** | **Domain 4** | **Domain 5** | **OVERALL** |
| --- | --- | --- | --- | --- | --- | --- |
| ASCEND4 | Low | Some concern | Low | Low | Low | Some concern |
| PROFILE 1014 | Low | Low | Low | Low | Low | Low |
| PROFILE 1029 | Low | Low | Low | Low | Low | Low |
| CONVINCE | Low | Some concern | Low | Low | Low | Some concern |
| ENSURE | Low | Low | Low | Some concern | Low | Some concern |
| EURTAC | Low | Low | Low | Some concern | Low | Some concern |
| FAST-ACT2 | Low | Low | Low | Low | Low | Low |
| HAN | Low | Low | Low | Some concern | Low | Some concern |
| IPASS | Low | Low | Low | Some concern | Low | Some concern |
| LUXLUNG3 | Low | Low | Low | Low | Low | Low |
| LUXLUNG6 | Low | Some concern | Low | Low | Low | Some concern |
| NEJ002 | Low | Low | Low | Some concern | Low | Some concern |
| OPTIMAL | Low | Some concern | Low | Some concern | Low | Some concern |
| WJTOG 3405 | Low | Low | Low | Some concern | Low | Some concern |
| ACHILLES | Low | Some concern | Low | Low | Low | Some concern |
| Yang | Some concern | Some concern | Low | Low | Low | Some concern |
| EXCLAIM-2 | Low | Some concern | Low | Low | Low | Some concern |
| PAPILLON | Low | Low | Low | Low | Low | Low |
| IMPOWER 150 | Low | Low | Low | Some concern | Low | Some concern |
| KEYNOTE 042 | Low | Some concern | Low | Some concern | Low | Some concern |
| KEYNOTE 189 | Low | Low | Low | Low | Low | Low |
| LIBRETTO-431 | Low | Low | Low | Low | Low | Low |

Domain 1: Bias due to randomization

Domain 2: Bias due to deviations from intended interventions

Domain 3: Bias due to missing data

Domain 4: Bias due to outcome measurement

Domain 5: Bias due to selection of the reported result

**Supplementary Table 4: Data comparing median progression-free survival (PFS) reported by original studies and their corresponding values reconstructed through the IPDfromKM method**

| **Study** | **Original**  **Median PFS months (95% CI)** | **Reconstructed**  **Median PFS months (95% CI)** |
| --- | --- | --- |
| ASCEND-4 | 8.1 (5.8-11.1) | 8.4 (5.8-11.1) |
| PROFILE 1014 | 7.0 (6.8-8.2) | 7.0 (6.8-8.2) |
| PROFILE 1029 | 6.8 (6.7-7.0) | 6.9 (5.7-7.1) |
| CONVINCE | 7.9 (6.5-10.2) | 10.3 (6.1-15.9) |
| ENSURE | 5.5 (NA-NA*) | 5.6 (5.5-8.2) |
| EURTAC | 5.2 (4.5-5.8) | 5.3 (4.5-6.0) |
| FAST-ACT2 | 6.0 (5.6-7.1) | 6.9 (5.0-7.5) |
| HAN | 5.7 (5.2-6.3) | 5.8 (3.9-7.8) |
| IPASS (EGFR subgroup) | NA (NA-NA*) | 6.1 (5.5-6.8) |
| LUX-LUNG3 | 6.9 (NA-NA*) | 7.1 (5.5-8.6) |
| LUX-LUNG6 | 5.6 (5.1-6.7) | 5.7 (5.1-7.0) |
| NEJ002 | 5.4 (NA-NA*) | 5.5 (4.9-6.1) |
| OPTIMAL | 4.6 (4.2-5.4) | 4.5 (4.1-5.4) |
| WJTOG 3405 | 6.3 (5.8-7.8) | 6.3 (5.8-7.6) |
| ACHILLES | 5.7 (4.2-7.2) | 5.8 (4.3-7.3) |
| Yang (post-hoc) | 8.2 (5.2-10.8) | 8.4 (5.2-10.9) |
| EXCLAIM-2 | 9.6 (7.2-11.4) | 9.8 (7.3-11.5) |
| PAPILLON | 6.7 (5.6-7.3) | 6.8 (5.6-7.4) |
| IMPOWER150 (post-hoc) | 5.8 (NA-NA*) | 6.4 (4.5-7.9) |
| KEYNOTE-042 | 6.2 (4.4-8.5) | 6.4 (4.2-8.6) |
| KEYNOTE-189 | 4.8 (4.6-9.0) | 4.7 (4.5-8.7) |
| LIBRETTO 431 | 11.2 (8.8-16.8) | 11.4 (8.9-16.9) |

*Not available

**Supplementary Table 5: Pairwise comparisons of pooled A) objective response rates, B) progression-free survival and C) overall survival between the actionable genomic alteration**

Pairwise comparisons of objective response rates between actionable genomic alteration were conducted assuming equal variances. Pairwise comparisons of progression-free survival and overall survival between actionable genomic alteration were performed using Cox proportional hazards regression.

**Sub-table 5a**

| *ALK* rearrangement | 1 |  |  |  |  |  |
| --- | --- | --- | --- | --- | --- | --- |
| Common *EGFR* mutation | .16 | 1 |  |  |  |  |
| Uncommon *EGFR* mutation | .70 | .70 | 1 |  |  |  |
| *EGFR* exon 20 insertion | .93 | .26 | .78 | 1 |  |  |
| *KRAS* mutation | .10 | .45 | .24 | .15 | 1 |  |
| *RET* fusion | .05 | .006 | .04 | .06 | .005 | 1 |
|  |  |  |  |  |  |  |
|  | *ALK* rearrange-ment | Common *EGFR* mutation | Uncommon *EGFR* mutation | *EGFR* exon 20 insertion | *KRAS* mutation | *RET* fusion |

**Sub-table 5b**

| *ALK* rearrangement | 1 |  |  |  |  |  |
| --- | --- | --- | --- | --- | --- | --- |
| Common *EGFR* mutation | <.001 | 1 |  |  |  |  |
| Uncommon *EGFR* mutation | .79 | .20 | 1 |  |  |  |
| *EGFR* exon 20 insertion | .11 | <.001 | .31 | 1 |  |  |
| *KRAS* mutation | .09 | .54 | .43 | .004 | 1 |  |
| *RET* fusion | <.001 | <.001 | <.001 | <.001 | <.001 | 1 |
|  |  |  |  |  |  |  |
|  | *ALK* rearrange-ment | Common *EGFR* mutation | Uncommon *EGFR* mutation | *EGFR* exon 20 insertion | *KRAS* mutation | *RET* fusion |

**Sub-table 5c**

| *ALK* rearrangement | 1 |  |  |  |  |
| --- | --- | --- | --- | --- | --- |
| Common *EGFR* mutation | .003 | 1 |  |  |  |
| Uncommon *EGFR* mutation | .40 | .73 | 1 |  |  |
| *EGFR* exon 20 insertion | .57 | .74 | .75 | 1 |  |
| *KRAS* mutation | <.001 | <.001 | .006 | <.001 | 1 |
|  | *ALK* rearrange-ment | Common *EGFR* mutation | Uncommon *EGFR* mutation | *EGFR* exon 20 insertion | *KRAS* mutation |

**Supplementary Fig 1: Objective response rates pooled by actionable genomic alteration according to region (Asia vs Global), 20 trials**

**Supplementary Fig 2: Objective response rates pooled by actionable genomic alteration according to pemetrexed or non-pemetrexed chemotherapy, 20 trials**
